# Applying the German S2k-Guideline for Diagnosis and Treatment of Spondylodiscitis—A 5-Year Retrospective Evaluation of Patients without Neurological Symptoms

**DOI:** 10.3390/diagnostics14111098

**Published:** 2024-05-25

**Authors:** Moritz Kolster, Alexander Hönning, Wiebke Käckenmester, Janet Goy, Axel Ekkernkamp, Nikolai Spranger

**Affiliations:** 1 Department of Trauma and Orthopaedic Surgery, BG Klinikum Unfallkrankenhaus Berlin gGmbH, 12683 Berlin, Germany; axel.ekkernkamp@ukb.de (A.E.); nikolai.spranger@ukb.de (N.S.); 2 Centre for Clinical Research, BG Klinikum Unfallkrankenhaus Berlin gGmbH, 12683 Berlin, Germany; alexander.hoenning@ukb.de (A.H.); wiebke.kaeckenmester@ukb.de (W.K.); 3 Department of Radiology and Neuroradiology, BG Klinikum Unfallkrankenhaus Berlin gGmbH, 12683 Berlin, Germany; janet.goy@ukb.de

**Keywords:** spondylodiscitis, vertebral infection, vertebral osteomyelitis, spondylitis, spine surgery

## Abstract

Spondylodiscitis is a rather rare condition with an annual incidence of 1–7 per 100,000. Thus, empirical data on the treatment of this disease are limited. In 2020, the first German guideline for the diagnosis and treatment of spondylodiscitis was published. In a 5-year retrospective analysis, we examined the patient collective, the current diagnosis and treatment strategy, and the effect of Magnetic Resonance Imaging (MRI) diagnostics on therapeutic decisions of a consecutive monocentric cohort of 66 patients without neurological symptoms. The majority of the patients were male (55%) with a mean age of 74 years. Non-operative therapy was found to be associated with short-term treatment success in 54 (82%) of the patients. In 12 patients, who underwent surgical therapy, MRI diagnostics and clinical findings were equally important for the decision to perform a surgery. Patients treated operatively stayed for an average of 33.6 (±12.9) days in the hospital and thus significantly longer than non-operatively treated patients with 22.2 (±8.0) days. The in-house standard of care did not essentially deviate from the guideline’s recommendations. Future research should address early detection of the need for surgical therapy, and immediate anti-infective treatment appropriate to the detected pathogen.

## 1. Introduction

Spondylodiscitis is a rare infectious disease, precisely described as an osteomyelitis of the vertebral bodies and infection of the corresponding vertebral discs. The frequent lack of specific symptoms complicates the diagnostic procedure and leads to delayed diagnoses. In industrial countries, the annual incidence is estimated at 1–7 per 100,000 [1], with an increase in recent decades. This increase has been attributed to improved diagnostics and the rise in life expectancy [2], which results in significantly higher age-standardized case numbers [3]. Still, vertebral osteomyelitis accounts for only 3–5% of all osteomyelitis cases [4]. The clinical picture lacks a specific pathognomy—Pott’s triad of gibbus, abscess, and paralysis is no longer considered relevant today—which often results in a prolonged diagnostic procedure [5]. However, studies show that early treatment is associated with a better outcome [6]. In contrast, the mortality rate is up to 20% when the condition remains untreated [7,8]. Patients with comorbidities and risk factors such as intravenous (IV) drug use, implants, or immuno-suppression are particularly affected [8]. While spondylodiscitis can be specific (e.g., tuberculosis, brucellosis, fungal infections), non-specific spondylodiscitis is much more common. Among the detected pathogens, multi-resistant bacteria are increasingly observed [9].

To date, there are only a few publications describing the epidemiology and treatment of spondylodiscitis in Germany. The Division of Septic and Reconstructive Surgery within the Department of Trauma and Orthopaedic Surgery at our hospital BG Klinikum Unfallkrankenhaus Berlin (ukb, Berlin, Germany) is specialized in infectious diseases of the musculoskeletal system. Due to the lack of a national guideline for the diagnosis and treatment of spondylodiscitis, the division established a treatment algorithm in 2014, according to which all patients with suspected spondylodiscitis are treated. The first national guideline on spondylodiscitis (S2k-guideline) was published in August 2020 under the lead of the specialist societies of the German Spine Society (DGW) and the German Society for Orthopaedics and Orthopaedic Surgery (DGOOC) [10]. Since then, a consensus-based treatment algorithm resting upon the best available evidence has existed in German-speaking countries.

However, the role of a follow-up Magnetic Resonance Imaging (MRI) on the further diagnostic and therapeutic procedure remains unclear [11,12]. Therefore, the primary aim of this study is to compare our current treatment strategy with the recommendations of the S2k-guideline and to investigate the impact of MRI diagnostics on further procedures. The patient collective will be characterized, and the diagnostic and treatment strategy will be evaluated by a retrospective analysis based on routinely collected health data. A comparison of the in-house standard with the S2k-guideline should also reveal overlaps and differences between the two treatment algorithms. Another aspect to be examined is the decision-making process regarding operative or non-operative treatment.

## 2. Materials and Methods

### 2.1. Study Design

This study was prospectively registered with the German Clinical Study Registry (Deutsches Register Klinischer Studien [DRKS]) with DRKS-ID DRKS00025740 and conducted in accordance with the Declaration of Helsinki 2013. The institutional review board of the Berlin Chamber of Physicians (Ärztekammer Berlin, Berlin, Germany, Eth-08/21) provided ethical approval for this study and waived the necessity for written consent. In the retrospective analysis, only routinely collected health data were taken from the hospital information system. The data were pseudonymized prior to data analysis.

**Figure 1:**
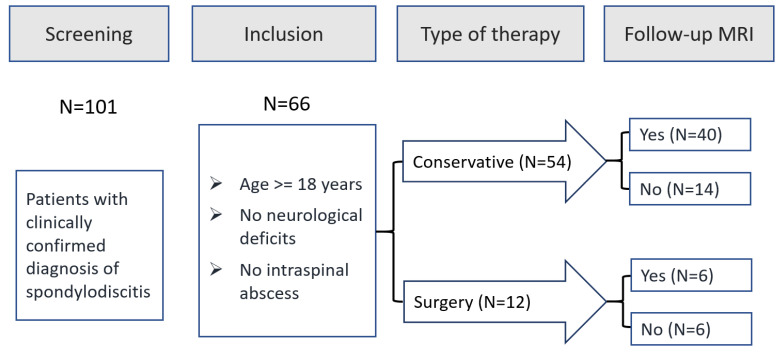
Study Flow Chart.

All patients with spondylodiscitis treated at our Department of Trauma and Orthopaedic Surgery between January 2015 (after implementation of the current treatment algorithm) and December 2019 (before publication of the s2k-guideline) were potentially eligible for study inclusion. Among 101 patients treated during this period, 66 patients met the predefined inclusion criteria and 46 patients underwent follow-up MRI (see Figure 1).

Patients who met the following inclusion criteria were eligible for study inclusion:

- Clinically confirmed diagnosis of spondylodiscitis
- Treatment of the condition at our hospital
- >=18 years of age

Patients with one or more of the following exclusion criteria were not eligible for study inclusion:

- Neurological deficits
- Intraspinal abscesses
- <18 years of age

### 2.2. Description of the In-House Treatment Algorithm

Patients with spondylodiscitis or highly suspected spondylodiscitis are treated based on a comprehensive treatment algorithm introduced in 2014 which is described in more detail below. 

The foundation for the diagnosis of spondylodiscitis is a summarized assessment of the patient’s medical history, clinical examination, laboratory parameters, and the conventional X-ray image in a standing position in two planes. A typical constellation of risk factors, clinical findings, elevated laboratory infection parameters, and possibly an abnormal X-ray leads to the working diagnosis of spondylodiscitis.

Further diagnostics require an initial documentation of vital signs and temperature in the emergency room as well as a blood sample to obtain a complete blood count, the c-reactive protein (CRP) as an inflammatory parameter, values to assess renal function, a gastroenterology/abdominal profile and, if applicable, thyroid values, followed by documentation of the patient’s medical history. Additionally, three pairs of blood cultures are taken separately, irrespective of the presence of fever. In cases of direct infections, such as after spinal surgery or injections, the clarification only serves to exclude other foci of infection. If the focus of infection is unclear, further diagnostics such as chest X-ray, urine analysis, sonography of the abdomen, echocardiography, and examination of dentate and otorhinopharyngeal structures are carried out.

Patients with a septic clinical appearance show fever, greatly increased infection laboratory parameters, and a poor general condition. This group of patients needs to receive an anti-infective treatment. If neurological deficits are present, emergency surgical treatment must be considered. Immediately after taking blood cultures, standardized antibiotic treatment with Cefuroxime and Fosfomycin is administered intravenously. Once bacterial resistance testing is completed, the antibiotics are adjusted to the microbiological findings. Patients with mild, chronic courses are only treated with antibiotics in accordance with the antibiogram once the pathogen has been detected. If no pathogen is detected in the first few days, the standardized antibiotic treatment begins. The same procedure applies in case of clinical deterioration. 

Symptomatic treatment with painkillers in accordance with the World Health Organization (WHO) analgesic ladder and patient factors (e.g., renal or liver dysfunction) is required. In severe cases with cardio-circulatory impairment, ICU treatment may be necessary.

In the first 24 h after admission, an MRI of the entire spine is performed to confirm the diagnosis. In case of contraindications to an MRI examination, a Computed Tomography (CT) scan with contrast medium should be performed.

During inpatient treatment, intravenous antibiotics are administered over 14 days, accompanied by routine monitoring of laboratory parameters as well as physiotherapy and analgesic therapy. As many patients are unable to care for themselves after hospital discharge, social services are involved to provide support in regaining independence. If new microbiological findings are obtained, anti-infective therapy might be adapted in consultation with colleagues from the microbiology department. Before discharge, the antibiotic treatment is changed to oral administration, which must be continued for at least four weeks.

A successful therapy describes a combination of clinical improvement (less pain, better mobilization, stable cardio-circulatory situation, no fever) and a decrease in the inflammatory parameters (leukocytes and CRP). Additionally, a follow-up MRI (complete spine without contrast medium) helps to assess potential new foci of infection, abscesses, and a change in local findings. If the imaging reveals a significant aggravation, non-operative treatment needs to be re-evaluated. 

A change from conservative to operative therapy is possible at any time and will be considered in case of the occurrence of neurological deficits, aggravating pain, increase in clinical infection symptoms and/or radiographic worsening of vertebral instability.

Discharge is planned in close consultation with the patient, relatives, and social services, if possible, to the patient’s home. Alternatively, further treatment in the geriatric departments or care facilities is possible. A follow-up examination is arranged six months after discharge, including MRI diagnostics.

### 2.3. Statistical Analysis

The statistical analysis was carried out descriptively in an exploratory manner. The sample size of the five-year documentation period from January 2015 to December 2019 was deemed sufficiently large to gain reliable and meaningful results. Depending on the scale level, absolute (*n*) and relative (%) frequencies, arithmetic mean value (mean), standard deviation (SD), as well as minimum and maximum were reported. To compare selected demographic variables and clinical parameters between non-operatively and operatively treated patients, a *t*-test for independent samples was calculated in case of continuous variables, and a Wilcoxon–Mann–Whitney test in the absence of a normal distribution. At the categorical scale level, the parameters were tested for significant differences at the significance level alpha = 0.05 using a Pearson’s chi-square test or, in the case of a cell frequency < 5, using a Fisher’s exact test. For clarity and readability, individual characteristics of some items were combined into categories (e.g., Charlson Comorbidity Index, localization of the infection, further treatment). All statistical analyses were carried out using SPSS V.27 (IBM, Armonk, NY, USA).

## 3. Results

### 3.1. Baseline Characteristics

The average age of the 30 female (45.5%) and 36 male (54.5%) patients was 73.7 (±9.7) years. Only one patient was younger than 58 years. Fifty-four (82%) of the 66 patients were treated conservatively, while 12 patients (18%) converted from initial non-operative to operative therapy. Neither the Charlson Comorbidity Index nor the inflammatory parameters (CRP and leukocytes) differed to a relevant extent between the conservatively treated and surgically treated groups on admission. Pathogens were detected significantly more often (*n* = 7, 58.3%) in the group of surgically treated patients (χ^2^ = 4.753; *p* = 0.029) than in the patients treated conservatively (*n* = 14, 25.9%). Most frequently (73%), the inflammation was localized in the lumbar spine. In about 4 out of 10 cases, the focus of infection was unclear. Urogenital infections were predominant among the remaining cases (see Table 1). Regarding comorbidities, there were no relevant differences between conservatively and surgically treated patients (see Appendix A Table A1).

### 3.2. Conversion from Non-Operative to Operative Therapy

In 12 (18%) of the 66 patients, conversion from conservative to surgical treatment was considered necessary. Six of the operated patients underwent follow-up MRIs, the other half only had initial MRIs on admission. Overall, MRI diagnostics and clinical findings were considered equally important for the decision to perform surgery. In 50% of the cases, the leading reason for conversion was the MRI and the secondary reason was based on the clinical findings; in the other half, the clinical findings were the primary basis for the conversion to surgery and the MRI findings were the secondary reason. Equally, when MRI follow-up examinations were performed, the findings of these MRIs were the leading reason for surgery in half of the patients (three out of six) and the clinical findings were the leading reason in the remaining three patients (see Table 2).

### 3.3. Short-Term Patient Outcome after Conservative/Surgical Therapy

**Figure 2:**
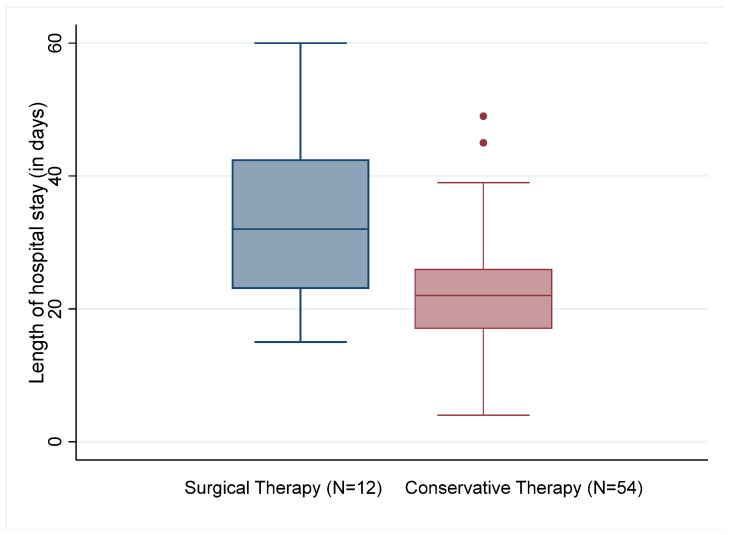
Length of hospital stay by type of therapy.

Of the 66 patients evaluated, two of the conservatively treated patients died (3.7%). None of the patients who underwent surgery died during the hospital stay. The surgically treated patients stayed in the hospital for 33.6 (±10) days on average and thus significantly longer than the patients who were treated conservatively with an average of 22.2 (±8.0) days (t(62)) = 2.908, *p* = 0.005; see Figure 2). Although antibiotics were administered over a longer time in patients who had undergone surgery (29.7 ± 15.5 days vs. 23.5 ± 18.7 days), the difference was not statistically significant.

The inflammation parameters did not show any relevant differences between groups. In both groups, inflammation parameters improved significantly between admission and discharge; the decrease was marginally more pronounced in the surgically treated patients. The proportion of patients with CRP values > 50 mg/L fell from 62.1% to 33.3%, while the proportion of patients with >10,000 leukocytes per microliter fell from 43.9% to 12.1%. 

After discharge from the hospital, both groups of patients were treated similarly. Around half of the patients were (re)transferred to a nursing home, around a third of the conservatively treated patients and half of the operated patients were treated on an outpatient basis, and around a tenth were subsequently transferred to (geriatric) inpatient care or died (see Table 3).

### 3.4. Comparison of the In-House Standard with the S2k Treatment Guideline

The treatment standard applied in our clinic since 2014 does not differ from the guideline’s recommendations in any of the key aspects (see Table 4). Deviations are caused by the lack of evidence, which is also the reason why the guideline is consensus-based and not evidence-based. At the time of the creation of the in-house treatment algorithm it seemed wise to include a broad spectrum of diagnostic steps to confirm the diagnosis and find the infective focus. Both diagnostic algorithms regard the medical history as an important tool for the early diagnosis of spondylodiscitis. The guideline further addresses the clinical examination, which primarily includes the clarification of neurological deficits and focus localization. In both algorithms, laboratory blood tests, blood cultures, and a focus search complement the clinical examination. Frequent infection sites are the lungs, the urogenital tract, and the oral cavity. Because this patient collective usually shows multiple comorbidities, an echocardiogram is used to clarify signs of endocarditis. The in-house standard of care only differs from the guideline’s algorithm in an additional ear, nose, and throat (ENT) examination and an abdominal ultrasound in the diagnostic process. The diagnostic focus lies on the pathogen detection, which enables an antibiotic treatment appropriate to the present pathogen. A biopsy or puncture should be performed according to both the guideline and the in-house standard if the blood culture analysis does not detect a specific pathogen.

The in-house algorithm and the guideline’s recommendations are largely consistent when it comes to treatment strategies. In critically ill patients with a septic circulatory condition, both recommend immediate, empirical, and ideally intravenous antimicrobial treatment. Pathogen-specific treatment according to the antibiogram is preferred in all other cases. Regarding the duration of anti-infective treatment, the guideline states: “Currently, there is no clear consensus regarding the duration of antibiotic therapy. From the existing retrospective studies, only expert opinions can be derived [...].” The in-house standard specifies two weeks of intravenous therapy followed by at least four weeks of oral therapy, which is consistent with clinical data and current treatment concepts [13]. 

The guideline recommends routine control of blood samples including the inflammation parameters CRP and leukocytes as well as daily documentation of pain and neurological findings. Subsequently, the treatment success should be assessed based on the patient’s overall condition. An evaluation based solely on infection parameters is explicitly not recommended. According to the guideline, repeated MRI/CT imaging is only recommended in the event of treatment failure and/or neurological deficits. The in-house algorithm includes a follow-up MRI after 14 days to provide a paraclinical treatment control, especially regarding possible intraspinal abscess formation or stenosis.

The algorithm clearly shows that a broad clinical infrastructure is required in diagnosing and treating vertebral infections. The recommended transfer to spine centers is therefore necessary. 

The monitoring of treatment success is controversially discussed in the literature and lacks specific directions in the S2k-guideline. However, the common focus lies on symptom relief (pain and infection symptoms) along with improved mobilization. The decrease in laboratory infection parameters is of secondary importance. The surgical treatment itself is not part of the algorithm examined here.

**Table 4:** Comparison of the in-house standard with the S2k treatment guideline.

Stage	S2k-Guideline Recommendation	Expert Consensus Achieved	In-House Standard
Preclinical	Treatment in a hospital with a specialized center for spinal disorders In case of suspected instability/neurological deficit: immobilization on a vacuum mattress (according to the guideline for polytrauma [14]) In septic patients: intensive medical transport and circulatory stabilization (according to the guideline for sepsis [15])	Consensus Strong consensus Strong consensus	Complies with the guideline
Anamnesis	Neurological deficit? Pain (duration, intensity, NRS)? Vital parameters (BP, pulse, temperature, GCS) Need for medication (pain and circulation)? Previous interventions on the spine (surgery or infiltration) and in general (teeth, implants, etc.) Previous treatment of pain or previous diagnostics Risk factors/comorbidities/red flags (B-symptoms, immunosuppressants, implants, alcohol/tobacco, pathogen exposure, travel, obesity)	Strong consensus for further investigation/exclusion in case of spinal complaints and corresponding medical history Strong consensus for documentation of NRS at admission and daily thereafter	Complies with the guideline
Physical Examination	Neurological deficit Maximum point of pain Protective posture? Psoas sign?	Strong consensus for assessment of neurological status at admission and daily thereafter	Complies with the guideline
Diagnostics	Blood sampling: small blood count and CRP (no ESR) Conventional X-ray of the affected spinal section in standing position in 2 planes Possibly qSOFA score Total spine MRI if possible, with contrast (if contraindicated: CT with contrast; in severe neurological deficits: emergency MRI) Pathogen detection preferably before the start of antibiotic therapy (3x blood culture, puncture/biopsy from disc, and endplate for microbiology and histology); incubation at least 14 days; possibly pathogen PCR Focus search: chest X-ray, general surgical laboratory parameters, echocardiography (with gram-positive pathogen), urine analysis, dental status	Strong consensus Strong consensus Strong consensus Strong consensus	In addition to the guideline: Abdominal ultrasound ENT consultation Implant clarification (pacemakers, prostheses, etc.)
Therapy	Non-operative: only slight/mild clinical symptoms, no neurological deficit, no/minor bone destruction, patient’s wish/contraindications for surgery Operative: (pre-)sepsis, relevant neurological deficit, intraspinal abscess, ventral/paravertebral abscess >2.5 cm, failure of conservative therapy, progressive instability/deformation Emergency surgery in severe neurological deficits		Complies with the guideline
Anti-infective therapy	Anti-infective therapy: In septic patients: start of an empirical intravenous antibiotic treatment covering S. aureus, Enterobacterales, and streptococci In patients with mild symptoms: wait for microbiological results and antibiogram-appropriate intravenous antibiotic therapy Duration of antibiotic therapy: 6 weeks orally or intravenously; possibly longer in case of risk factors, severe course, no improvement; in specific cases of spondylodiscitis: 9 months	Strong consensus for antibiotic coverage of the most common pathogens Strong consensus for antibiotic treatment for at least 6 weeks	Septic patients: empirical antibiotic therapy with Fosfomycin/Cefuroxime; otherwise: pathogen-adjusted 2 weeks IV, then 4 weeks PO Oral antibiotic therapy with Levofloxacin/Rifampicin or pathogen-adjusted
Pain treatment	Multimodal pain therapy according to national guidelines for non-specific lower back pain Pain therapy according to WHO’s analgesic ladder In case of persistent pain > 6 NRS, pain therapy co-treatment	Strong consensus Strong consensus Strong consensus	Pain therapy according to the WHO scheme Co-treatment by pain specialists if necessary
Mobilization	Axis-aligned mobilization and back-friendly behavior Consideration of orthotic treatment	Strong consensus Strong consensus	Axis-aligned mobilization No orthotic treatment
Follow-up	Routine blood samples for monitoring of leukocytes and CRP X-ray for follow-up checks Imaging only in case of treatment failure, neurological deficits Case conference with microbiology, radiology, infectious diseases, spinal surgery in case of treatment failure Referral to pain therapy in case of pain lasting > 3 months	Strong consensus: Imaging only in case of treatment failure Strong consensus: Expand search for the source in case of treatment failure Strong consensus: Referral to pain therapy in case of pain lasting > 3 months Strong consensus for case conferences	Routine blood samples (leukocytes, CRP, kidney, electrolytes) Conventional X-ray for acute symptoms Follow-up MRI after 14 days of intravenous antibiotic treatment

Abbreviations: NRS—Numeric Rating Scale, BP—blood pressure, GCS—Glasgow Coma Score, CRP—C-reactive protein, ESR—erythrocyte sedimentation rate, qSOFA—quick Sequential Organ Failure Assessment, MRI—Magnetic Resonance Imaging, CT—Computed Tomography, PCR—polymerase chain reaction, ENT—Ear Nose Throat, IV—intravenous, PO—peroral, WHO—World Health Organization.

## 4. Discussion

Our cohort’s demographic characteristics are consistent with previous studies. The slightly increased risk of men having spondylodiscitis in our study (55% vs. 45%) was also reported by Issa et al. [1] (51% males), Jeong et al. [16] (58% males), Krogsgaard et al. [17] (58% males), and Akiyama et al. [18] (59% males). Moreover, the average age of our study patients of 73.7 years is consistent with relevant studies that found the highest incidence of spondylodiscitis among those aged 60–79 years [17] and greater than 70 years [19]. Our in-hospital mortality rate (3.0%; 2 out of 66 patients) was rather low compared to previous studies reporting in-hospital mortality ranging from 2.1% [1], 3% [17], and 6% [18] to 12% [20]. The fatal cases in the conservatively treated group (3.7%; 2 out of 54 patients) are most likely due to the standard of non-operability of severely ill patients.

Due to a lack of reliable studies on the duration and use of antibiotic therapy, the guideline only recommends a treatment period of at least six weeks for non-specific spondylodiscitis. In the case of specific spondylodiscitis or persistent symptoms, the antibiotic therapy might be extended. Equivalently, in an open-label, randomized controlled trial comparing antibiotic treatment for 6 weeks versus 12 weeks in patients with spondylodiscitis, it was concluded that 12 weeks of antimicrobial treatment offer no clinical advantage over 6 weeks of treatment [21]. The antibiotic therapy recommended in the guideline is essentially based on the procedure commonly used in septic surgery, as described by the PRO IMPLANT Foundation, among others [13]. Under intravenous antibiotic treatment, a significant reduction was achieved in half of the patients with severely elevated CRP values, and in two thirds of the patients with severely elevated leukocyte counts. Laboratory parameters were not documented regularly after the patient was discharged with continued oral antibiotics.

Despite extensive focus searches and attempts to identify the pathogen, the guideline’s estimation was confirmed: The infection focus was identified in only 60% of the patients, and the pathogen detection was successful in around 30%. Accordingly, antibiotic treatment was often empirical (most common pathogen: Staph. aureus with approx. 60% [22]). The administration of Cefuroxime and Fosfomycin proved to be effective, so that conservative treatment was successful in 82% of the cases. Treatment success was measured by a decrease in inflammation parameters combined with an improvement in clinical findings and regressive or at least unchanged MRI findings. The role of follow-up MRI in the clinical decision making is much debated without final conclusions [11,23]. Conversion to surgical treatment was only necessary in 12 patients (i.e., in 18% of cases), which is considerably lower than the conversion rate of 53% observed in a high-volume tertiary care center in Germany [19]. The decision for surgical therapy was never based on a single factor, but rather on a combination of clinical, paraclinical, and radiological findings.

With an average of 24.2 days, the in-hospital length of stay was shorter compared to most previous studies [17,19,24,25], but longer than in a study from the United States [1] with an average of only 9.2 days. The length of stay did not include subsequent inpatient treatment (e.g., in secondary care hospitals and geriatric departments), and increased with secondary conversion to surgery to around 33 days. The authors of the US study attributed the substantially shorter in-hospital time to the extensive use of home intravenous antibiotic therapy (OPAT: outpatient parenteral antimicrobial therapy) as well as the general effort to reduce the in-hospital time in the US. In the context of an increasing medical economization, outpatient intravenous antibiotic therapy might also be considered in Germany [26].

Data gathered in this study help to sharpen diagnostic and therapeutic algorithms as patient profiles are clearer now and guideline recommendations validated. Further research is scheduled to analyze the radiological algorithm and define focus-specific treatment groups.

We acknowledge several limitations of the present study. One limitation of this study is the nature of a retrospective analysis, in which no parameters tailored to the research question can be collected; instead, data documented in the treatment routine that were captured for a different purpose need to be used. Patient-oriented outcome measures (PROMs) such as pain levels, functional outcomes, or patient satisfaction with the course of recovery, which may be used as relevant criteria for longer-term treatment success in a prospective survey, can therefore not be considered. Moreover, the rather small sample size, particularly in the group of patients who underwent an operative therapy, only allows limited conclusions to be drawn and does not rule out the possibility that differences occurred by chance. Another limitation is that the exclusion of patients with neurological deficits or intraspinal abscesses, which are usually associated with a more severe form of spondylodiscitis, may introduce selection bias. For this reason, our conclusions can only refer to the patient population included in our study. Finally, the present study is limited by the lack of follow-up data to further assess the outcomes of patients treated with spondylodiscitis. Patient-oriented outcome measures such as functional outcomes or patient satisfaction with the course of recovery cannot be considered. Therefore, the long-term success of the conservative and surgical treatment strategies cannot be evaluated based on the current data.

Spondylodiscitis is a rare but serious disease that should be diagnosed and treated in specialized spine centers. Even the initial diagnostic procedure must be conducted in a multidisciplinary manner and may require therapeutic interventions from various specialties. The same applies to anti-infective decisions and the interdisciplinary treatment of a seriously ill patient population. Given the congruence between the S2k-guideline and the in-house standard of care, we consider the non-operative treatment strategy employed in our clinic in accordance with the S2k-guideline’s recommendations. In future research, a large, multicenter trial with the documentation of PROMs is needed to validate the results found in this study. Moreover, an early detection of the need for surgical therapy, and the appropriate anti-infective treatment should be addressed.

## Figures and Tables

**Table 1:** Baseline characteristics by type of therapy.

Variable	All Patients (*N* = 66)	Conservative (*N* = 54)	Surgery (*N* = 12)
Age, Mean ± SD	73.7 ± 9.7	74.6 ± 12.9	73.3 ± 8.1
Gender, *n* (%)			
Male	36 (54.5%)	27 (50.0%)	9 (75.0%)
Female	30 (45.5%)	27 (50.0%)	3 (25.0%)
Charlson Index, Mean ± SD	5.97 ± 3.25	6.13 ± 3.35	5.25 ± 2.77
Charlson Index, *n* (%)			
1–4	24 (36.4%)	19 (35.2%)	5 (41.7%)
5–8	27 (40.9%)	21 (38.9%)	6 (50.0%)
>8	15 (22.7%)	14 (25.9%)	1 (8.3%)
CRP uptake, *n* (%)			
Elevated >50 mg/L	41 (62.1%)	34 (63.0%)	7 (58.3%)
Leukocytes uptake, *n* (%)			
Elevated (>10,000)	29 (43.9%)	23 (42.6%)	6 (50.0%)
Detection of Pathogen, *n* (%) *	21 (31.8%)	14 (25.9%)	7 (58.3%)
Type of Pathogen, *n* (%)			
Staph. aureus	8 (38.1%)	7 (50.0%)	1 (14.3%)
Other pathogen	6 (28.6%)	2 (14.3%)	4 (57.1%)
Not documented	7 (33.3%)	5 (35.7%)	2 (28.6%)
Localization, *n* (%)			
Cervical spine	5 (7.6%)	4 (7.4%)	1 (8.3%)
Thoracic spine	13 (19.7%)	8 (14.8%)	5 (41.7%)
Lumbar spine	48 (72.7%)	42 (77.8%)	6 (50.0%)
Focus of infection, *n* (%)			
Indeterminate	26 (39.4%)	19 (35.2%)	6 (50.0%)
Urogenital	15 (22.7%)	14 (25.9%)	1 (8.3%)
Extremities	7 (10.6%)	7 (13.0%)	0
Pulmonary	5 (7.6%)	5 (9.3%)	0
Oral	5 (7.6%)	4 (7.4%)	1 (8.3%)
Implant	4 (6.1%)	2 (3.7%)	2 (16.7%)
Local	2 (3.0%)	2 (3.7%)	0
Abdominal	2 (3.0%)	1 (1.9%)	1 (8.3%)
Follow-up MRI, *n* (%)	46 (69.7%)	40 (74.1%)	6 (50.0%)

Abbreviations: Mean—Arithmetic Mean, SD—Standard deviation, *N*/*n*—Number of patients, %—Frequency, Staph.—Staphyolococcus, CRP—C-reactive protein, mg/L—milligrams per liter, MRI—Magnetic Resonance Imaging, * *p* < 0.05.

**Table 2:** Reasons for conversion from conservative therapy to surgery.

		All Patients (*N* = 12)	Follow-Up MRI (*N* = 6)	No Follow-Up MRI (*N* = 6)
Reasons for Conversion, *n* (%)				
Primary Reason	Secondary Reason			
Initial MRI	Clinical finding	3 (25.0%)		3 (50.0%)
Clinical finding	Initial MRI	3 (25.0%)		3 (50.0%)
Follow-up MRI	Clinical finding	3 (25.0%)	3 (50.0%)	
Clinical finding	Follow-up MRI	3 (25.0%)	3 (50.0%)	

Abbreviations: *N*/*n*—Number of patients, %—Frequency, MRI—Magnetic Resonance Imaging.

**Table 3:** Outcome by type of therapy.

Variable	All Patients (*N* = 66)	Conservative (*N* = 54)	Surgery (*N* = 12)
Hospital stay			
Length of stay, Mean ± SD *	24.2 ± 10.1	22.2 ± 8.0	33.6 ± 12.9
In-hospital mortality, *n* (%)	2 (3.0%)	2 (3.7%)	0
Labor parameters			
CRP at admission, Mean ± SD	106.2 ± 111.7	111.9 ± 116.2	80.5 ± 88.8
CRP at discharge, Mean ± SD	44.1 ± 38.5	44.4 ± 39.0	42.7 ± 37.5
CRP at admission (>50 mg/L), *n* (%)	41 (62.1%)	34 (63.0%)	7 (58.3%)
CRP at discharge (>50 mg/L), *n* (%)	22 (33.3%)	19 (35.2%)	3 (25.0%)
Leukocytes at admission, Mean ± SD	10.1 ± 4.1	10.1 ± 4.3	10.2 ± 3.1
Leukocytes at discharge, Mean ± SD	7.0 ± 2.9	7.1 ± 3.0	6.9 ± 2.6
Leukocytes at admission (>10,000), *n* (%)	29 (43.9%)	23 (42.6%)	6 (50.0%)
Leukocytes at discharge (>10,000), *n* (%)	8 (12.1%)	6 (11.1%)	2 (16.7%)
Duration of antibiosis iv, Mean ± SD	24.6 ± 18.2	23.5 ± 18.7	29.7 ± 15.5
Radiological diagnosis (Follow-up), *n* (%)			
Success of therapy **	19 (50.0%)	15 (46.9%)	4 (66.7%)
Failure of therapy	19 (50.0%)	17 (53.1%)	2 (33.3%)
Follow-up treatment, *n* (%)			
Nursing home	37 (56.1%)	31 (57.4%)	6 (50.0%)
Ambulant/post-hospital curative treatment	23 (34.8%)	17 (31.5%)	6 (50.0%)
Acute inpatient follow-up treatment	4 (6.1%)	4 (7.4%)	0
Deceased	2 (3.0%)	2 (3.7%)	0

Abbreviations: Mean—Arithmetic Mean, SD—Standard deviation, *N*/*n*—Number of patients, %—Frequency, CRP—C-reactive protein, mg/L—milligrams per liter, iv—intravenous, * *p* < 0.05. ** Decision based on a combined evaluation of inflammation parameters, clinical findings and MRI findings.

## Data Availability

The datasets analyzed within this study are available from the corresponding author on reasonable request.

## Appendix A

**Table A1:** Comorbidities by therapy.

Variable	All Patients (*N* = 66)	Conservative (*N* = 54)	Surgery (*N* = 12)
Psoas abscess, *n* (%)	32 (48.5%)	27 (50.0%)	5 (41.7%)
Carcinoma, *n* (%)	14 (21.2%)	12 (22.2%)	2 (16.7%)
Coronary heart disease, *n* (%)	30 (45.5%)	26 (48.1%)	4 (33.3%)
Renal insufficiency, *n* (%)	32 (48.5%)	27 (50.0%)	5 (41.7%)
Diabetes mellitus, *n* (%)	31 (47.0%)	23 (42.6%)	8 (66.7%)
Immunosuppressed, *n* (%)	8 (12.1%)	8 (14.8%)	0
Fever, *n* (%)	13 (19.7%)	12 (22.2%)	1 (8.3%)

Abbreviations: *n*—Number of patients, %—Frequency.
